# Sociodemographic Disadvantage, Living With a Smoker, and Health Risk Behaviors in Middle-Aged and Older Women

**DOI:** 10.1093/geroni/igaa057.1083

**Published:** 2020-12-16

**Authors:** Carole Holahan, Charles Holahan, Sangdon Lim, Yen Chen, Daniel Powers

**Affiliations:** 1 University of Texas at Austin, Austin, Texas, United States; 2 The University of Texas at Austin, Austin, Texas, United States

## Abstract

Sociodemographic disadvantage places individuals at risk for an unhealthy lifestyle (Kushi et al., 2012; Shanker et al., 2010), as well as for exposure to second-hand household smoke (Gan et al., 2015; Zhang et al., 2012). However, the role of living with a smoker in the association between sociodemographic status and health behavior is unstudied. This study investigated the role of living with a smoker in partially explaining the link between sociodemographic disadvantage and physical inactivity and poor dietary behaviors. The study used limited access data from the Women’s Health Initiative Observational Study obtained from NHLBI. Participants were 83,597 women ranging in age from 49 to 81; 6038 participants lived with a smoker. Cross-sectional logistic regression analyses examined paths in the models; bias-corrected bootstrapped confidence intervals tested indirect effects in probit analyses. Analyses controlled for age, ethnicity, marital status, and participants’ current smoking status. Results demonstrated a significant association (p < .001) between sociodemographic disadvantage (composite of low education and low income) and living with a smoker (OR = 1.74). The unstandardized indirect effects (CIs are in brackets) from sociodemographic disadvantage through living with a smoker to no exercise, no walking, high percent dietary fat, and low servings of fruits and vegetables through living with a smoker were statistically significant (.023 [.019, .028], .026 [.023, .033], .041 [.037, .047], and .032 [.027, .036], respectively). These findings illustrate the need to address multiple non-smoking health risk behaviors in household smoking interventions for disadvantaged families. This project was supported by the NIH/NCI (R03CA215947).

